# Associations Between Childhood Trauma and the Age of First-Time Drug Use in Methamphetamine-Dependent Patients

**DOI:** 10.3389/fpsyt.2021.658205

**Published:** 2021-03-26

**Authors:** Cui Huang, Qiuyu Yuan, Ling Zhang, Lei Wang, Shu Cui, Kai Zhang, Xiaoqin Zhou

**Affiliations:** ^1^Chaohu Hospital, Anhui Medical University, Hefei, China; ^2^School of Mental Health and Psychological Sciences, Anhui Medical University, Hefei, China; ^3^Anhui Psychiatric Center, Anhui Medical University, Hefei, China

**Keywords:** methamphetamine, age of first-time drug use, minor, childhood trauma, family environment

## Abstract

Childhood trauma is related to substance use disorder; however, few studies have examined the relationship between childhood trauma and the age at which the drug was first used. The aim of this study was to investigate the relationship between childhood trauma and the age of first-time drug use among methamphetamine-dependent patients. Moreover, we analyzed the characteristics of adverse family environment associated with severe childhood trauma and the risk factors for starting drugs in minors. A baseline interview was conducted with 110 participants who were in detoxification, including demographic information, past substance use, and age of first-time drug use. The participants' childhood trauma experience before 18 years of age was evaluated using the simplified version of the Childhood Trauma Questionnaire (CTQ-SF). The Chinese version of the Family Environment Scale (FES-CV) was used to assess the family environment of methamphetamine-dependent patients. Among 110 non-injecting methamphetamine-dependent patients, nearly half (*n* = 48, 43.6%) had moderate and severe childhood trauma. Correlation analysis showed that the age of first-time drug use negatively correlated with emotional abuse (*r* = −0.32, *p* < 0.01) and physical abuse (*r* = −0.27, *p* < 0.01). The age of first-time drug use negatively correlated with conflict (*r* = −0.20, *p* < 0.05) and independence (*r* = −0.22, *p* < 0.05) of family environment, but positively correlated with intellectual-cultural orientation (*r* = 0.28, *p* < 0.01). Additionally, childhood trauma factors significantly correlated with many indexes of family environment, especially cohesion (*r* = −0.45, *p* < 0.01), conflict (*r* = 0.49, *p* < 0.01), and independence (*r* = 0.33, *p* < 0.01). Additionally, the regression model showed that when emotional abuse increased by one point, the age of first-time drug use was 0.69 years earlier. These findings suggest that a detrimental family environment can aggravate childhood trauma, and the experience of childhood emotional or physical abuse may be an effective predictor of early drug use among methamphetamine-dependent patients.

## Introduction

According to the 2020 World Drug Report released by the United Nations Office on Drugs and Crime, approximately 35.6 million people suffer from substance-related disorders globally. The use of amphetamines, especially methamphetamine, is increasing in parts of Asia and North America, with adolescents and young adults accounting for the largest share of users (1). According to the Drug Situation Report released by the Chinese government in 2019, methamphetamine drug has the largest number of abusers in China, with 1.186 million abusing methamphetamine, accounting for 55.2% of the 2.148 million existing drug users (2).

Drug abuse leads to a variety of serious consequences, such as severe depression (3), and even suicide attempts (4); drug abusers gradually become socially vulnerable and marginalized, such as unemployed and homeless. It must be noted that the social, economic, and public health burden associated with substance-related disorders, especially methamphetamine dependence, is enormous (5, 6). Moreover, worldwide, the average age at which a drug is used for the first time is reported to be relatively low, most of which begins when the individuals are underage (7–10). Therefore, initial age of drug use is becoming younger, and the issue of drug abuse among teenagers and even students needs to be urgently addressed (11). As there are no approved drugs for methamphetamine-dependent disorders currently, psychotherapy and symptomatic treatment are the main approaches (12).

The epidemiological causes of drug abuse are complex and multifactorial, and many risk factors, including childhood trauma, are related to substance use (13, 14). High levels of traumatic childhood experiences have been observed in people with substance-related disorders (15). In a study of 113 adult opioid addicts treated with buprenorphine, 80.5% reported experiencing at least one type of childhood trauma (16). Several adverse psychological and behavioral consequences of childhood abuse include depression, post-traumatic stress disorder, anxiety, attention deficit hyperactivity disorder, and conduct disorder (17). Additionally, a meta-analysis concluded a causal relationship between childhood abuse, including emotional and physical abuse, drug use, mental disorders, attempted suicide, risky behaviors, and sexually transmitted diseases (18). Moreover, in a 5-year cohort study, childhood sexual abuse was found to increase the risk of teenagers starting using injecting drugs (19).

Family environment is the most important part of childhood; an unfavorable family environment has a negative impact on children's growth (20). Parents' substance dependence behavior is closely related to children's awful childhood experiences, and children exposed to parental alcoholism are more likely to suffer from various forms of abuse, neglect, and family dysfunction.

Psychotherapy is an important research topic. Current studies have focused on childhood trauma and drug use, but there are few reports on the relationship between childhood trauma and the age of first-time drug use. Drug abuse generally starts from non-injection, gradually forms drug dependence, and finally moves to the injection mode (21–23). The transition from non-injecting drug use to injecting drug use greatly increases blood-borne diseases such as HIV (24–26); conversely, rates of high-risk injection and sexual activity were lower in people who started using drugs later (27). Therefore, our goal was to explore whether childhood trauma leads to an increase in the age of the first-time non-injecting drug use, and the impact of the family environment on methamphetamine-dependent patients. This provides a theoretical basis for treatment in the field of psychology. In this way, drug users who have experienced childhood trauma can have an opportunity to intervene appropriately in reducing the number of relapses, restore social function, and reduce social burden.

## Materials and Methods

### Participants

The participants were recruited from a compulsory rehabilitation center in the Anhui Province, China. Individuals who met the following criteria were included in this study: (1) adults (≥18 years old); (2) met the Diagnostic and Statistical Manual of Mental Disorders, 5th edition (DSM-5) criteria for methamphetamine dependence; (3) were non-injecting drug users, and (4) can provide informed consent. Concurrently, participants who met any of the following exclusion criteria were excluded: (1) suffering from severe mental illness, such as schizophrenia, depressive disorder, and so on; (2) those who have used psychoactive substances in the past month; (3) those who have comorbidity with serious organic diseases, including cerebrovascular disease, cardiovascular disease, and neurodegenerative disease.

**Figure 1:**
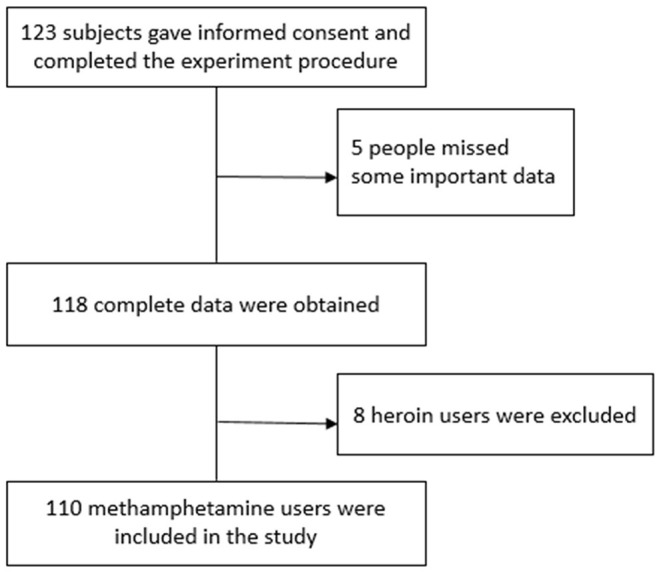
CONSORT flow diagram.

After screening, 123 patients were eligible to participate in the study. Of the 13 excluded samples, eight were heroin users and five had incomplete data. As a result, 110 methamphetamine-dependent patients were included in this study (Figure 1).

This study was approved by the Ethics Committee of Chaohu Hospital of Anhui Medical University (Ethics number: 201901-kyxm-02). All research procedures were in line with the Helsinki Declaration. First, we explained the purpose, method, and process of the study to the participants in detail to ensure that the participants understood the study. After obtaining informed consent from the participants, we conducted the research using a questionnaire, and did not use any invasive or harmful examination, or medication.

### Measures

A socio-demographic questionnaire was designed to collect the enrolled patients' socio-demographic information. The questionnaire mainly included age, sex, race, education status, and marital status; all the information was filled in by the participants themselves.

The childhood trauma of amphetamine users was subsequently evaluated using the simplified version of the Childhood Trauma Questionnaire (CTQ-SF), a 28-item retrospective self-report questionnaire that briefly screened the history of mental and physical abuse and neglect in childhood and adolescence before the age of 18 years. The original English version of the CTQ-SF was compiled by Professor Bernstein and his colleagues and was later translated into Chinese by Zhao et al. (28, 29). The Chinese version of the CTQ-SF has good reliability and validity (30, 31). CTQ-SF is also applicable to adult drug abusers (32).

Additionally, to explore the family environmental factors influencing childhood trauma, we used a Chinese version of the Family Environment Scale (FES-CV); it comprises 90 items classified into 10 areas: cohesion, expressiveness, conflict, independence, achievement orientation, intellectual-cultural orientation, active-recreational orientation, moral-religious emphasis, organization, and control (33). The FES-CV has been widely used with good reliability and validity (34).

### Statistical Analysis

First, we described the socio-demographic characteristics of the participants, and then divided them into minor and adult groups according to the age of the first-time drug use. The data were expressed as mean ± standard deviation. The Kolmogorov-Smirnov single-sample test was used to evaluate the normal distribution of continuous variables. According to the results of the normality test, Pearson correlations or Spearman correlations were used to determine the correlations between each family environment variable and each childhood trauma subtype of amphetamine users. Then, the childhood trauma of the minor and adult groups were compared. Independent sample *t* test or the Mann Whitney *U* test were used to compare the two groups depending on the normal distribution of the continuous variables. Additionally, the chi-square test was used to compare classified variables. Finally, linear regression and binary logistic regression were applied to obtain more valuable information. Statistical analyses were performed using IBM SPSS21.0. Statistical significance was set at *p* < 0.05, and all reported *p* values were bilateral.

## Results

### Social Demographic Characteristics of Methamphetamine Users

The sociological characteristics and drug abuse data are presented in Table 1. A total of 110 methamphetamine-dependent patients were included in this study, and none of them used injections. The participants were all Han Chinese adult males, with an average age of 30.7 ± 3.6 years. Among them, 46 (41.8%) were unmarried, 35 (31.8%) were married, and 29 (26.4%) were divorced. Additionally, 65 (59.1%) had urban household registration, and 45 (40.9%) had rural household registration. The average length of education of the participants was 8.27 ± 3.03 years. The majority (*n* = 76, 69.1%) reported having a junior high school education or below, some (*n* = 33, 30.0%) reported having high school education, and only one had completed his college. There was a significant difference between the adult and minor groups. Participants who started using methamphetamine before adulthood were likely to be less educated, unmarried, and lived in cities.

**Table 1:** Social demographic characteristics of methamphetamine dependent patients.

	**Total (*n* = 110)**	**Minor (*n* = 21)**	**Adult (*n* = 89)**	***t*/*Z*/λ**	***p***
Age (years)	30.70 ± 3.60	28.20 ± 3.20	31.30 ± 3.50	−3.74	0.00
Education (years)	8.30 ± 3.00	6.80 ± 2.40	8.60 ± 3.10	−2.53	0.01
Marital status				12.16	0.00
Unmarried	46 (41.80%)	16 (76.00%)	30 (34.00%)		
Married	35 (31.80%)	2 (10.00%)	33 (37.00%)		
Divorced	29 (26.40%)	3 (14.00%)	26 (29.00%)		
Place of household registration				110.00	0.00
Urban areas	65 (59.10%)	15 (71.00%)	50 (56.00%)		
Rural areas	45 (40.90%)	6 (29.00%)	39 (44.00%)		
Relapse times	0.70 ± 1.00	1.00 ± 1.40	0.60 ± 1.00	1.76	0.08
Frequency of drug abuse per month	13.80 ± 11.80	16.00 ± 12.30	13.30 ± 11.70	−0.97	0.33

### Association Between Childhood Trauma, Family Environment and the Age of First-Time Drug Use

Nearly half (48, 43.6%) of the 110 participants had moderate and severe childhood trauma. The average age of initial drug use was 22.6 ± 4.2 (oldest, 32 years; youngest, 14 years). According to Table 2, the Pearson correlation analysis shows that the age of first-time drug use negatively correlated with the total score of childhood trauma (*r* = −0.24, *p* < 0.05), emotional abuse (*r* = −0.32, *p* < 0.01), and physical abuse (*r* = −0.27, *p* < 0.01). The age of first-time drug use negatively correlated with conflict (*r* = −0.20, *p* < 0.05) and independence (*r* = −0.22, *p* < 0.05) of family environment, but positively correlated with intellectual-cultural orientation (*r* = 0.28, *p* < 0.01). Additionally, childhood trauma factors significantly correlated with many indexes of family environment, especially cohesion (*r* = −0.45, *p* < 0.01), conflict (*r* = 0.49, *p* < 0.01), and organization (*r* = −0.29, *p* < 0.01).

**Table 2:** Correlation between childhood trauma, family environment and age of first-time drug use.

***n* = 110**	**Age of first drug use**	**Childhood trauma**	**Emotional abuse**	**Physical abuse**	**Sexual abuse**	**Emotional neglect**	**Physical neglect**
Age of first drug use	–	−0.24^*^	−0.32^**^	−0.27^**^	−0.09^**^	−0.16	−0.03
Cohesion	0.03	−0.45^**^	−0.28^**^	−0.28^**^	−0.17	−0.37^**^	−0.38^**^
Expressiveness	0.07	−0.22^*^	−0.14	−0.24^*^	−0.03	−0.23^*^	−0.07
Conflict	−0.20^*^	0.49^**^	0.47^**^	0.41^**^	0.18	0.38^**^	0.24^*^
Independence	−0.22^*^	0.33^**^	0.19	0.33^**^	0.09	0.29^**^	0.15
Achievement orientation	−0.02	−0.12	−0.04	−0.09	−0.09	−0.09	−0.09
Intellectual-cultural orientation	0.28^**^	−0.13	−0.12	−0.16	0.06	−0.14	−0.03
Active-recreational orientation	0.12	−0.21^*^	−0.19^*^	−0.15	−0.08	−0.18	−0.10
Moral-religious emphasis	−0.05	−0.15	−0.06	−0.08	−0.06	−0.17	−0.08
Organization	0.14	−0.29^**^	−0.21^*^	−0.24^*^	−0.13	−0.20^*^	−0.22^*^
Control	0.12	−0.11	−0.10	−0.13	0.04	−0.16	0.01

* *p < 0.05*,

** *p < 0.01*.

### The Differences in Childhood Trauma Between Minor Group and Adult Group

The results of the Mann-Whitney *U* test (Table 3) show that the minor group had more severe childhood trauma (*Z* = −2.15, *p* = 0.03), especially emotional (*Z* = −3.10, *p* = 0.00) and physical abuse (*Z* = −2.77, *p* = 0.01) than the adult group. Additionally, there was no significant difference in relapse times and frequency of drug abuse between the two groups (Table 1).

**Table 3:** The difference of childhood trauma between minor group and adult group.

	**Minor (*n* = 21)**	**Adult (*n* = 89)**	***Z***	***p***
Childhood trauma	42.90 (12.15)	37.13 (7.91)	−2.151	0.031
Emotional abuse	8.10 (2.28)	6.62 (1.75)	−3.099	0.002
Physical abuse	8.29 (4.36)	6.13 (1.80)	−2.772	0.006
Sexual abuse	5.71 (1.27)	5.81 (1.64)	−0.189	0.850
Emotional neglect	11.76 (4.62)	9.70 (3.64)	−1.899	0.058
Physical neglect	9.05 (3.85)	8.88 (2.87)	−0.115	0.908

### Quantitative Analysis of Factors Influencing Age of First-Time Drug Use

Based on a further analysis of the age of first-time drug use, we found that under age methamphetamine-dependent patients had a higher level of childhood trauma than those who started using methamphetamine in adulthood.

We conducted a linear regression analysis between the age of first-time drug use and childhood trauma factor scores, and entered it stepwise; emotional abuse was retained in the regression model. The regression model showed that when emotional abuse increased by 1 point, the age of first-time drug use was 0.69 years earlier. The age of first-time drug use and the number of childhood traumas were analyzed using regression analysis; for each additional type of childhood trauma, the age at first-time drug use was 0.95 years earlier. Finally, the binary logistic regression between first-time time drug abuse in adulthood and family environmental factors showed that conflict was a risk factor for starting drugs in minors (*p* = 0.04) (data not shown).

## Discussion

In this study, we examined the relationship between childhood trauma and the age at which non-injecting drug was used for the first-time time among Chinese methamphetamine-dependent patients. It was found that the age of first-time drug use of methamphetamine-dependent patients experiencing severe childhood trauma, especially emotional abuse or physical abuse, will be earlier, with more childhood trauma subtypes. The in-depth quantitative analysis conducted in our study shows that, in the regression model, for each additional score of emotional abuse, the age of first-time drug use will be 0.69 years earlier, and for each additional trauma subtype, the age of the first-time drug use will be 0.95 years earlier. Our study found no correlation between physical neglect and age of first-time drug use, even though the incidence of physical neglect was high. After exploring the causes of childhood trauma our findings suggest that family environmental factors are strongly associated with the level of childhood trauma and the age of first-time drug use.

Previous research has shown that childhood trauma can lead to early drug use (7, 35), which is consistent with the results of our study. However, existing studies have focused on drug users who inject the drug, while this study focused on drug users who use non-injecting drugs. Drug abuse usually begins with non-injecting drug use and gradually transforms into injecting the drug under the influence of a variety of complex factors (36–38). Therefore, from this point of view, our study on the age of first-time drug use among non-injecting drug users is more sensitive, persuasive, and practical than the research on injecting drug use.

Our study observed that emotional and physical abuse was associated with first-time drug use at younger ages before adulthood. Emotional abuse refers to the long-term and inappropriate emotional reaction of the caregivers or others to children, such as malicious refusal, isolation, intimidation, or the use of insulting, satirical and discriminatory language to treat children. Physical abuse refers to actual or potential physical harm to children caused by the rude and inappropriate behavior of caregivers or others. Physical and emotional abuse in childhood has a negative effect on mental health in adulthood (39, 40). A correlation was found between childhood trauma and emotional disorders (41), and emotional regulation is found to play an intermediary role in childhood trauma led substance abuse (42). Early childhood stress interferes with the maturation of brain networks associated with cognitive and emotional processes, which adversely affect cognitive and emotional processes during adolescence (43). Additionally, the brain structure of people who experienced childhood trauma changed accordingly, including reduction of the caudate nucleus (44), hippocampus (45), and amygdala (46). Similar results were obtained in animal models of childhood trauma, such as loss of hippocampal volume, and destruction of dendritic structures (47). Childhood trauma can affect HPA axis activity, which may be related to post-traumatic depression (48). There is a significant correlation between childhood trauma and low cortisol arousal levels (49), resulting in pro-inflammatory state, which plays an important role in the development of emotional disorders in adulthood (50). Traumatic events can lead to negative emotions, which persist over time, are difficult for individuals to manage, and may lead to the use of drugs for self-treatment (51, 52). Especially in individuals who do not have enough emotional regulation strategies to tolerate strong negative emotions, substance use may become a repetitive and maladaptive coping mechanism. In presence of multiple traumatic experiences, substance use may intensify, and individuals exposed to traumatic events may turn to substance use, to mitigate the long-term negative effects of exposure to trauma (53). This mechanism of mood disorders leads to early drug use in individuals with severe childhood trauma. Furthermore, these results are similar to our study's results, which show that longer the years of education, or the older the age of first-time drug use, lower the number of relapses. Compared to people with low levels of education, people with higher education know more emotional regulation strategies, while older people are better at emotional regulation than teenagers.

Our study found that the more subtypes of childhood trauma, the earlier the age of first-time drug use. A previous national study in the UK found a significant cumulative relationship between trauma and mental illness, and the more types of trauma, the higher the probability of mental illness (54). Early childhood abuse will physically change neurobiological development, excluding direct physical harm, have a negative impact on cognitive, emotional, and social growth, and lead to psychological, behavioral, and learning problems; these problems will continue throughout the whole life (55–57). A growing body of evidence suggests that the origins of most adult diseases are associated with childhood development and biological disorders. These early life experiences may affect the mental and physical health of adults, either through the accumulation of time or the biological embedding of adversity during sensitive developmental periods (58). Physical abuse in childhood may be an experience that changes neurobiological development and increases the risk of mental illness.

Although a high incidence of physical neglect among methamphetamine-dependent patients has been observed, the internal correlation with the age of first-time drug use could not be found. To investigate its reason, several previous studies had shown that the internal consistency of CTQ-SF 's physical neglect subscale is relatively low (31, 59, 60), and this phenomenon can be attributed to the cultural differences in the definition of physical neglect.

Our results show that family environment is closely related to the level of childhood trauma and the age of first-time drug use. Methamphetamine-dependent patients who experienced high levels of family conflict and independence, or low family cohesion and emotional expression, reported more severe childhood trauma. Cohesion and conflict are mainly reflected in the relationships among family members. In a low cohesion and high conflict family environment, family members lack communication and do not publicly express their views and emotions. Previous studies have shown that psychosocial background includes childhood trauma, maternal mental illness, maladaptive parenting styles, and dysfunctional parent-child relationships, all of which are recognized as contributing factors to the development of marginal characteristics (61). Borderline personality disorder has a mediating effect on drug abuse and childhood trauma, and there is a relationship between multiple childhood trauma and borderline personality (62–64). Additionally, childhood abuse has a cumulative effect, and those who have experienced more developmental abuse show significantly higher levels of marginal characteristics (65). Synchronously, borderline personality shows more impulsive behaviors, including alcoholism, drug abuse (66), gambling, and promiscuity (67). Conversely, positive affect and resilient coping in adulthood were both positively correlated with positive family environment and negatively correlated with childhood trauma (68). In adulthood, when faced with adversity, individuals with poor ability to resist adversity will develop maladaptive behavior, including drug abuse. Additionally, previous studies have shown that family history of drug and alcohol abuse is closely associated with childhood trauma (35), and that maternal alcohol abuse is significantly associated with all types of childhood trauma except physical abuse, while physical abuse is associated with paternal alcohol abuse (69). Meanwhile, the family history of drug use is related to the earlier age of first drug use, and childhood physical trauma plays an intermediary role in the relationship between the family history of substance use problems and the age at first-time drug use (7).

There are some limitations to our research. First, this study uses a cross-sectional survey, so causality cannot be inferred. Second, our study is based on the participants in the cultural background of the Han nationality in China, which may not be completely representative of other cultural backgrounds in the world. Third, the sample size of the minor group of amphetamine users in this study was not large enough, leading to the restriction of discovering some meaningful indicators. Finally, the CTQ-SF is a self-retrospective reporting tool that may not accurately reflect the level of trauma exposure, and recall bias may also affect our results, especially those who frequently take drugs are more likely to recall the history of childhood abuse.

## Conclusion

In short, childhood trauma is a strong predictor of the age at which drug is used for the first time, and the more severe childhood trauma is, the younger the age of first-time drug use is. Trauma interventions for drug users and early trauma screening and treatment for children and adolescents may reduce drug abuse throughout life. Therefore, public health policies need to address this specifically in order to reduce the chances of potential drug users becoming drug users.

## Data Availability Statement

The raw data supporting the conclusions of this article will be made available by the authors, without undue reservation.

## Ethics Statement

The studies involving human participants were reviewed and approved by Ethics Committee of Chaohu Hospital of Anhui Medical University. The patients/participants provided their written informed consent to participate in this study. Written informed consent was obtained from the individual(s) for the publication of any potentially identifiable images or data included in this article.

## Author Contributions

The manuscript was designed and written by author CH. Data was collected by CH, QY, LZ, LW, and SC, analyzed by CH and KZ, and verified by XZ. All authors read and agreed to the final manuscript.

## Conflict of Interest

The authors declare that the research was conducted in the absence of any commercial or financial relationships that could be construed as a potential conflict of interest.
